# Cholecystoduodenal Fistula and Urosepsis in A Febrile Emergency Department Patient: A Case Report

**DOI:** 10.5811/cpcem.21291

**Published:** 2025-01-16

**Authors:** Amna Nawaz, Denise Elizondo, Bushra Hussein, Rebecca Theophanous

**Affiliations:** * Duke University School of Medicine, Department of Emergency Medicine, Durham, North Carolina; † Durham Veterans Affairs Healthcare System, Durham, North Carolina

**Keywords:** Fever, staghorn calculus, cholecystoduodenal fistula, point-of-care ultrasound, case report

## Abstract

**Introduction:**

Point-of-care ultrasound (POCUS) is a rapid bedside tool, particularly in undifferentiated emergency department patients. Point-of-care ultrasound can investigate potential intra-abdominal infections in febrile patients, especially in the elderly, who often present atypically without abdominal pain or localizing symptoms.

**Case Report:**

We highlight the important POCUS findings of cholecystoduodenal fistula and staghorn calculus in a febrile, elderly patient with dementia.

**Conclusion:**

Early recognition of cholecystoduodenal fistula and staghorn calculus using POCUS can expedite appropriate antibiotic and interventional treatment for improved patient outcomes.

## INTRODUCTION

Point-of-care ultrasound (POCUS) is a useful clinical tool in guiding diagnostic and management plans, particularly in undifferentiated emergency department (ED) patients.^1^,^2^ The benefits of ultrasound include the ability to perform the exam bedside in unstable patients, reduced ED and hospital costs, decreased lengths of stay, and improved patient safety avoiding ionizing radiation.^2^ Recognition of intra-abdominal POCUS findings is important in guiding appropriate treatment in patients requiring early surgical or interventional procedures, for example in acute cholecystitis, choledocholithiasis, nephrolithiasis, and urinary retention.^3^–^11^

Cholecystoduonedal fistula and staghorn calculi can lead to serious complications if not recognized and treated early. These unusual findings can be visualized bedside with POCUS.^6^–^11^ Aside from a few older case reports, literature on this topic is sparse. We describe the characteristic findings that are critical for emergency physicians to recognize in order to expedite treatment.^6^–^11^ We also describe how POCUS can be used in undifferentiated febrile patients to expedite or narrow the diagnosis, including in older adults who often have insidious etiologies and atypical presentations without abdominal pain or localizing symptoms.^12^–^14^

We present a case report of a febrile, elderly patient with dementia. We describe the important POCUS findings of cholecystoduodenal fistula causing *Clostridium perfringens* bacteremia and staghorn calculus causing urosepsis, and we compare these findings to computed tomography (CT) imaging.

## CASE REPORT

An 89-year-old elderly man with hypertension, diabetes mellitus, benign prostatic hypertrophy, seizure disorder, and prior subdural hemorrhage presented to the ED via ambulance after two unwitnessed falls at his skilled nursing facility. Paramedics placed him in a cervical collar, and he received acetaminophen for fever. On ED arrival, the patient was confused and not oriented to self, place, or time. Vital signs included blood pressure 92/49 millimeters of mercury, heart rate 100 beats per minute, oxygen saturation 98% on room air, respiratory rate 16 breaths per minute, and temperature of 39.1° degrees Celsius. On physical exam, the patient was confused, with a Glasgow Coma Scale of 14. His lungs were clear, and abdomen was soft, non-tender, and non-distended with normal bowel sounds. He had a right periorbital contusion. His neurological exam was limited due to his underlying cognitive deficits and confusion, but he moved all extremities with full strength and sensation.

On traumatic workup, chest and pelvis radiographs were normal. Non-contrast computed tomography of his head and cervical spine revealed a non-operative, right nondisplaced zygomatic arch fracture and chronic right subdural hematoma. Laboratory tests revealed a leukocytosis of 18.2x10^9^ per liter (L) (reference range 3.2–9.8 x10^9^/L), hemoglobin of 9 grams per deciliter (g/dL) (13.7–17.3 g/dL), hematocrit of 28.9% (39–49%), normal electrolytes, elevated blood urea nitrogen of 29 milligrams (mg) per dL (mg/dL) (7–20 mg/dL), creatinine of 1.1 mg/dL (0.6–1.3 mg/dL), glucose of 128 mg/dL (70–140 mg/dL), aspartate aminotransferase of 51 units (U)/L (15–41 U/L), alanine aminotransferase of 26 U/L) (15–50 U/L), total bilirubin of 0.7 mg/dL (0.4–1.5 mg/dL), conjugated bilirubin of 0.3 mg/dL (0.1–0.6 mg/dL), alkaline phosphatase of 76 U/L (24–110 U/L), lactate of 1.4 millimoles (mmol)/L (0.6–2.2 mmol/L), and pH 7.41. His coronavirus disease test was negative.

Due to the patient’s fever and altered mental status, while awaiting CT imaging transport and performance, emergency physicians performed a biliary POCUS exam that showed a gallbladder full of air and sludge with shadowing and a duodenal fistula at the medial aspect of the gallbladder view (Supplemental Video 1). Gallbladder thickness was normal (0.27 centimeters); the common bile duct was not visualized due to shadow artifact. Renal POCUS exam also revealed a left intrarenal staghorn calculus (hyperechoic stone with posterior shadowing) and moderate hydronephrosis (dilation of renal pelvis and calyces) (Supplemental Video 2).

**Image 1:**
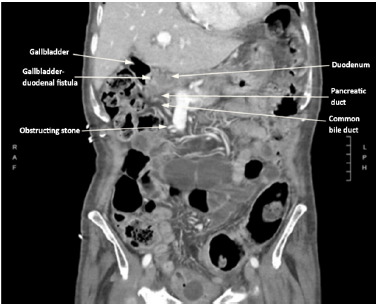
Computed tomography coronal view of a gas-containing gallbladder with fistulous connection to the proximal duodenum. There are common bile duct and main pancreatic duct dilatation due to an obstructing stone within the distal common bile duct.

**Image 2:**
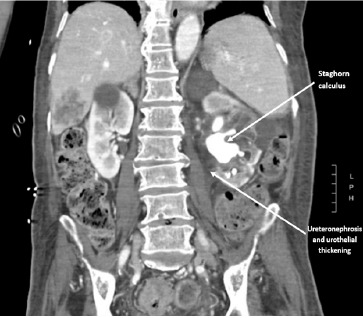
Computed tomography coronal view of a large left staghorn calculus with significant hydroureteronephrosis and urothelial thickening.

An abdominal/pelvic CT confirmed 1) a gas-containing gallbladder with fistulous connection to the proximal duodenum (Image 1), and 2) left staghorn calculus with significant hydroureteronephrosis and urothelial thickening (Image 2). He also had partial small and large bowel obstructions due to a new colorectal mass concerning for primary malignancy, with rectosigmoid inflammation and an adjacent contained perforation, and liver metastases.

Urology was consulted since the patient could not urinate. A urethral stricture was dilated, and a Foley catheter was placed. His urinalysis had greater than 182 white and red blood cells per high power field (hpf) (reference range <5/hpf and <3/hpf), 2+ protein, 3+ blood, 3+ leukocytes, (reference range negative), negative nitrite, 5 squamous cells, and gross blood from traumatic Foley placement. The patient was treated for urosepsis with intravenous (IV) fluids and IV piperacillin-tazobactam and vancomycin, and he was admitted to the internal medicine service.

**CPC-EM Capsule**

What do we already know about this clinical entity?

*Point-of-care ultrasound (POCUS) as a bedside tool can investigate potential intra-abdominal infections in febrile patients*.

What makes this presentation of disease reportable?

*We highlight the important POCUS findings for cholecystoduodenal fistula and staghorn calculus in a febrile, elderly patient*.

What is the major learning point?

*Cholecystoduodenal fistula is an abnormal gallbladder and duodenal connection with air. Staghorn calculus is a hyperechoic structure with shadowing in the kidney*.

How might this improve emergency medicine practice?

*Early recognition of intra-abdominal disease using POCUS can expedite appropriate antibiotic and interventional treatment for improved patient outcomes*.

Two days later, both blood cultures grew *C perfringens* (presumed from biliary/perforated gastrointestinal source), and the urine culture grew *Enterococcus faecalis*. After goals of care discussions with the patient’s family, due to his frail condition, severe dementia, and likely new diagnosis of metastatic colon cancer with intestinal obstruction and contained bowel perforation, invasive therapeutic intervention with endoscopic retrograde cholangiopancreatography and surgical intervention of the cholecystoduodenal fistula and staghorn calculi were deferred. The patient was treated with IV antibiotics for seven days and discharged back to his facility with goals to prioritize comfort and avoid further hospitalizations.

## DISCUSSION

Point-of-care ultrasound can expedite diagnosis and guide treatment decisions, especially in unstable patients with hypotension or sepsis.^1^,^2^ Because intra-abdominal infections can present atypically in older adults, as was evident in our patient, POCUS can help evaluate potential sources including biliary or renal pathology.^1^,^2^,^12^,^13^ Very few cases of spontaneous gallbladder-duodenal fistula resulting from stone erosion through the gallbladder wall into the duodenum have been described.^6^–^10^ Gallbladder-duodenal fistula is important to diagnose because serious complications and illness from bacterial invasion and leakage into the biliary system and intra-abdominal cavity can occur if not treated quickly with antibiotics and surgery. The most common bilioenteric fistula is cholecystoduodenal, occurring in 40% of cases.^6^–^10^

Risk factors include female gender, old age, large stones, and recurrent cholangitis.^6^–^10^ Malignancy, peptic ulcer disease, hydatid disease, and iatrogenic injury can also form aberrant connections between the gallbladder and enteric tract.^8^,^9^ Point-of-care ultrasound and CT findings may reveal air and mixing of biliary/intestinal contents, with an abnormal fistula between the gallbladder wall and duodenum, and visualization of air bubbles traveling between the gallbladder and duodenum, as seen in our patient.^6^,^7^,^10^ Point-of-care ultrasound can also demonstrate a finding called “dirty shadowing,” which is caused by intra-abdominal gas or air that reflects the sound waves and appears as reverberations against a solid organ, which is abnormal.^1^,^10^

Regarding our patient’s infected staghorn calculus, POCUS was used to diagnose intrarenal nephrolithiasis and hydronephrosis.^11^ On POCUS, staghorn calculi are large hyperechoic or bright white structures with posterior acoustic shadowing (vertical line of darkness inferior to the object on the screen) that conform to the shape of the renal pelvis. These stones can cause hydroureteronephrosis, which is seen as dilation of the renal pelvis and the ureter.^1^ Again, recognizing these POCUS findings can yield improved patient outcomes with early antibiotics and surgical removal.^1^,^2^ Stone analysis can guide treatment.

Struvite or calcium carbonate apatite staghorn calculi are associated with urease-producing bacteria, requiring antibiotics and expulsion therapy.^14^ In contrast, metabolic stones composed of calcium phosphate, uric acid, calcium oxalate, or cystine require changes in urine alkalinization and fluid rehydration to promote expulsion and prevent reformation.^14^ Staghorn calculi can cause pyelonephritis and sepsis, especially in dehydration or decreased renal flow. Urologic interventions such as stone removal, intraureteral stenting, or percutaneous nephrostomy tube placement are critical and time-sensitive treatments in septic patients and can be expedited with renal POCUS at the bedside.^11^,^14^

This case also highlights the importance of a thorough workup and evaluation of fever in the undifferentiated ED patient without early anchoring, especially in those with cognitive deficit.^12^,^13^,^15^,^16^ Because older adults have diminished physiologic reserve, it is imperative to minimize diagnostic delays, as delayed or incorrect treatment can cause permanent morbidity including end-organ damage and death, particularly in sepsis.^12^,^13^,^15^ In addition to infection, the differential for fever in older adults should include drug reactions, polypharmacy, malignancy, autoimmune disorders, and hematologic pathology.^15^,^16^ Workup includes a thorough history and physical exam, with assistance from family members or facility staff.^13^,^14^ Diagnostics include blood tests with a blood gas, lactate, and blood and urine cultures if there is concern for sepsis.^15^,^16^ Diagnostic imaging includes chest radiograph and bedside abdominal ultrasound to expedite diagnosis and treatment, with a low threshold for CT imaging. Lastly, physicians should halt non-essential home medications to avoid symptom-masking and initiate fluid resuscitation, antibiotics, and antipyretics as indicated.^15^,^16^

## CONCLUSION

Emergency physicians should incorporate point-of-care ultrasound in undifferentiated febrile patients to optimize correct treatment and expedite patient care, particularly in older adults who present atypically. We highlight the unique POCUS findings for cholecystoduodenal fistula and intrarenal staghorn calculus. Both findings typically require surgical or interventional treatments in addition to IV antibiotics and, thus, should be identified early to prevent serious complications and mortality.

## Supplementary Information

Supplemental Video 1 Biliary point-of-care ultrasound longitudinal view of the gallbladder full of air and sludge with shadowing and duodenal fistula at the medial aspect of the gallbladder. Unable to visualize the common bile duct.

Supplemental Video 2 Renal point-of-care ultrasound longitudinal view of a left intrarenal staghorn calculus with shadowing and moderate hydronephrosis.
